# Ultrasound-guided interventions with augmented reality *in situ* visualisation: a proof-of-mechanism phantom study

**DOI:** 10.1186/s41747-019-0129-y

**Published:** 2020-02-04

**Authors:** Nadja A. Farshad-Amacker, Till Bay, Andrea B. Rosskopf, José M. Spirig, Florian Wanivenhaus, Christian W. A. Pfirrmann, Mazda Farshad

**Affiliations:** 1 Radiology, Balgrist University Hospital, Forchstrasse, 340, 8008 Zurich, Switzerland; 2 Incremed AG, Lenghalde 5, 8008 Zurich, Switzerland; 3 Department of Orthopaedics, Balgrist University Hospital, University of Zurich, Forchstrasse 340, 8008 Zurich, Switzerland

**Keywords:** Augmented reality, Holography, Phantoms (imaging), Punctures, Ultrasonography

## Abstract

**Background:**

Ultrasound (US) images are currently displayed on monitors, and their understanding needs good orientation skills. Direct overlay of US images onto the according anatomy is possible with augmented reality (AR) technologies. Our purpose was to explore the performance of US-guided needle placement with and without AR *in situ* US viewing.

**Methods:**

Three untrained operators and two experienced radiologists performed 200 US-guided punctures: 100 with and 100 without AR *in situ* US. The punctures were performed in two different phantoms, a leg phantom with soft tissue lesions and a vessel phantom. Time to puncture and number of needle passes were recorded for each puncture. Data are reported as median [range] according to their non-normal distribution.

**Results:**

AR *in situ* US resulted in reduced time (median [range], 13 s [3–101] *versus* 14 s [3–220]) and number of needle passes (median [range], 1 [1–4] *versus* 1 [1–8]) compared to the conventional technique. The initial gap in performance of untrained *versus* experienced operators with the conventional US (time, 21.5 s [3–220] *versus* 10.5 s [3–94] and needle passes 1 [1–8] *versus* 1 [1, 2]) was reduced to 12.5 s [3–101] *versus* 13 s [3–100] and 1 [1–4] *versus* 1 [1–4] when using AR *in situ* US, respectively.

**Conclusion:**

AR *in situ* US could be a potential breakthrough in US applications by simplifying operator’s spatial orientation and reducing experience-based differences in performance of US-guided interventions. Further studies are needed to confirm these preliminary phantom results.

## Key points

- A novel augmented reality (AR) *in situ* ultrasound (US) technique has been proposed.
- AR technology allows for a direct overlay of US images onto the according anatomy.
- Using AR *in situ* US, operators could be faster and need less needle passes for US-guided interventions.
- AR *in situ* US may reduce experience-based differences in performing US-guided interventions.

## Background

Although ultrasound (US) technology is radiation free and cost-effective, it is subject to experience dependency and highly dependent on the level of training [1, 2]. In some institutions, diagnostic and therapeutic joint injections are performed under fluoroscopic guidance [3–5], even if US guidance for most joints would be possible [6, 7]. The main believed reason is reduction of observer dependency [8], paying a trade-off in terms of radiation exposure. Further fluoroscopic guidance usually is performed by injecting iodined contrast media for control of needle positioning, with the associated risk of allergic reactions [9]. Thus, a radiation-free modality such as US, but with a higher user-reproducibility, is desired.

Augmented reality (AR) is increasingly gaining impact in medicine, particularly for guidance purposes since its introduction in the 1980s [10–12]. Some studies were recently published highlighting the use of AR in puncture guidance [10, 11, 13–17]. With AR headsets, computed visual information (holograms) are displayed in real time as an overlay of the user’s field of view of reality.

We investigated if US with AR technology would achieve an AR *in situ* US view with the US image as hologram, displayed at the exact anatomic location in reality, creating a direct sonographic view for the operator. We hypothesised that AR *in situ* US would decrease inter-operator variability in performance, by simplifying the challenge of spatial orientation.

## Methods

No institutional review board was needed for this prospective phantom study.

### AR *in situ* US

**Fig. 1:**
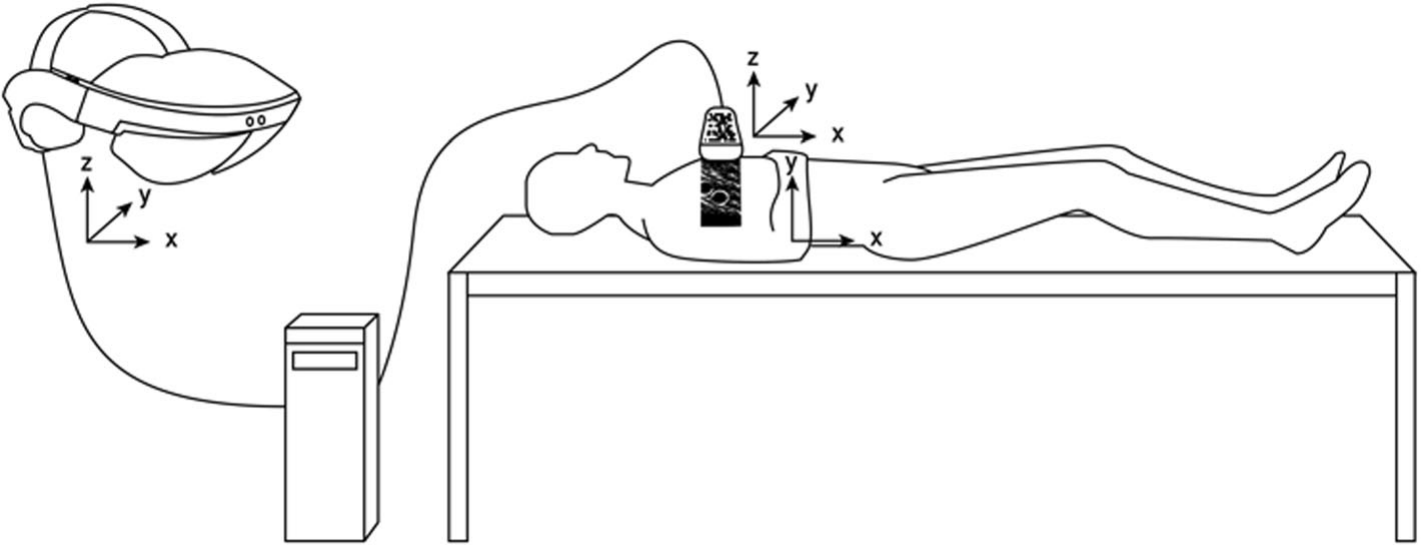
AR *in situ* US system with the AR headset, the computer, where the spatial mapping of the US image is calculated by the custom software and the transducer showing its physically attached tracking pattern. Not shown here: the standard US that is providing the US image data to the software on the computer. *AR* Augmented reality, *US* Ultrasound

The AR *in situ* US system is composed of a conventional US system (SuperSonic Aixplorer Ultimate, SuperSonic Imagine, Aix-en-Provence, France), custom developed software, industry-grade head-mounted AR displays (Microsoft Hololens, Redmond, Washington, USA) and physical extensions of the US transducer handles (Fig. 1).

The positioning of the US image is based on calculating a relative offset of the image to the tracking marker on the US probe with respect to the head-mounted device coordinate system. To perform the offset calculation, a calibration station was used with an exact cut-out for the probe head and whose geometrical dimensions are precisely known. The station also has a tracking marker, which is recognised by the head-mounted device. To properly register, the US image is placed relative to the station marker according to the known offset to the cut-out. From this positioning, the offset to the probe’s tracker marker is measured within the head-mounted device’s coordinate system.

By observing the tracking pattern attached to the US transducer, the system calculates the correct positioning of the US image in space. The same tracking pattern is used to first calibrate the system together with the interpupillary distance of the operator’s eyes. Using these coordinate system transformations and the information attained in the calibration step, the US image is transferred form the conventional US monitor to the AR headset. Thus, this transferred image is directly superimposed to the imaged anatomical structures. While scanning with the AR *in situ* US, the displayed superimposed image is update in real time as the operator moves the transducer, displaying the anatomical structures at their correct anatomical location in a 1:1 scale.

Additionally, to the superimposed imaged displayed in a 1:1 scale, the AR system also displays an enlarged version of the same image in an overhead position in the virtual three-dimensional space. This allows the operator to consult both the superimposed 1:1 image at the anatomical location, as well as the same, but larger, image when looking up.

The accurate image size is calibrated based on the scanning depth and width given by the external US system. Changes in any of these two parameters make recalibration of our system necessary. Usually, this information is readily available from the US devices. The US image is displayed on a two-dimensional plane with the correct scan depth and width. The plane is then rendered onto the head-mounted device display and is scaled, positioned, and rotated in such a way that it is perceived at the correct three-dimensional location. For example, for *in situ* visualisation, the plane is shown beneath the US probe where the image is generated.

### Phantom puncture

Three untrained operators (orthopaedic surgeons) without any experience in US and two experienced radiologists (with 9 and 11 years of training) performed 200 US-guided punctures with a linear transducer using a conventional US (SL 18-5, SuperSonic Aixplorer, SuperSonic Imagine, France) and with the AR *in situ* US (Microsoft Hololens and SuperSonic Aixplorer, SuperSonic Imagine, Aix-en-Provence, France).

**Fig. 2:**
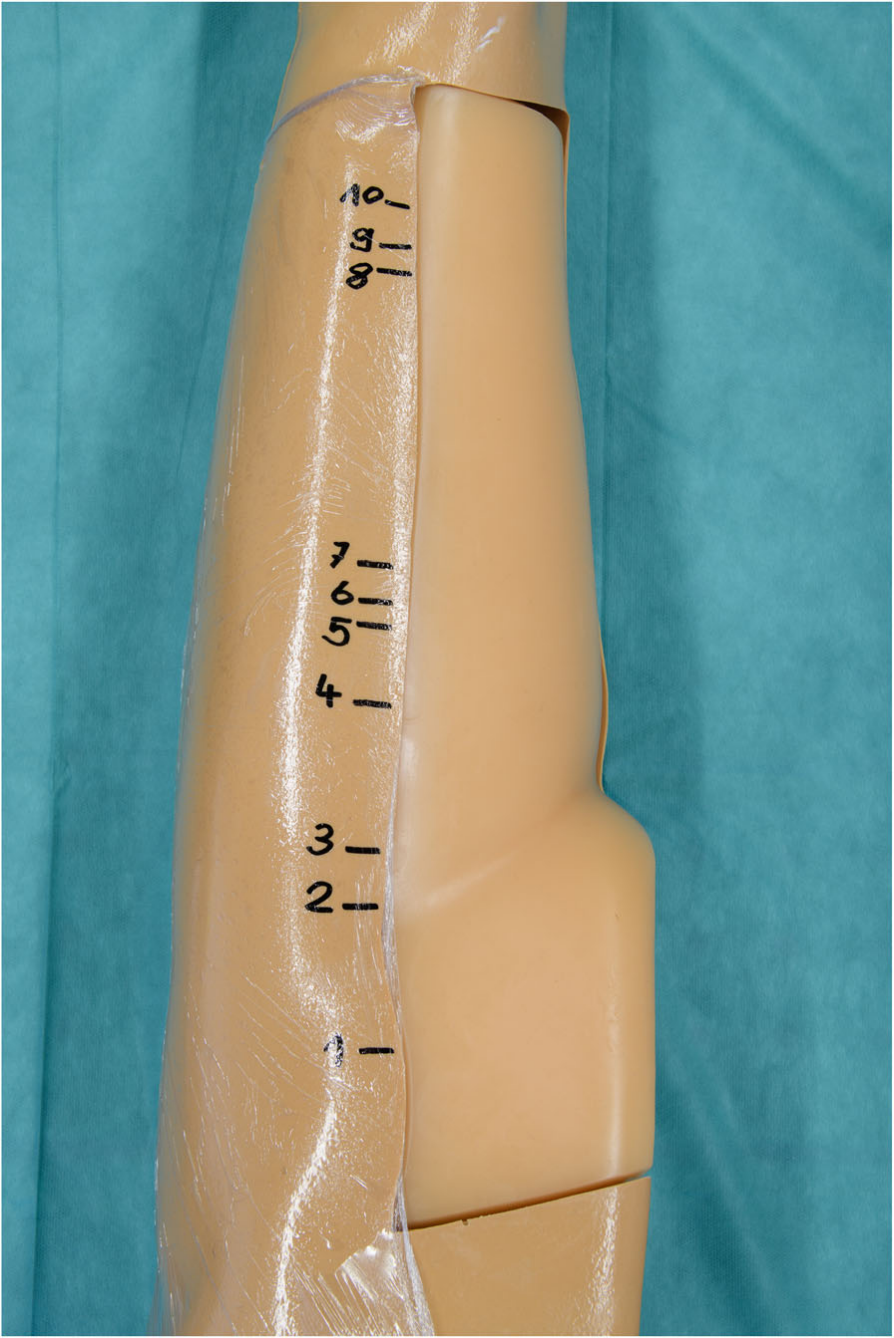
The prepared leg phantom. Ten out of 20 lesions were marked, so that each operator punctuated the same lesions in the same order

**Fig. 3:**
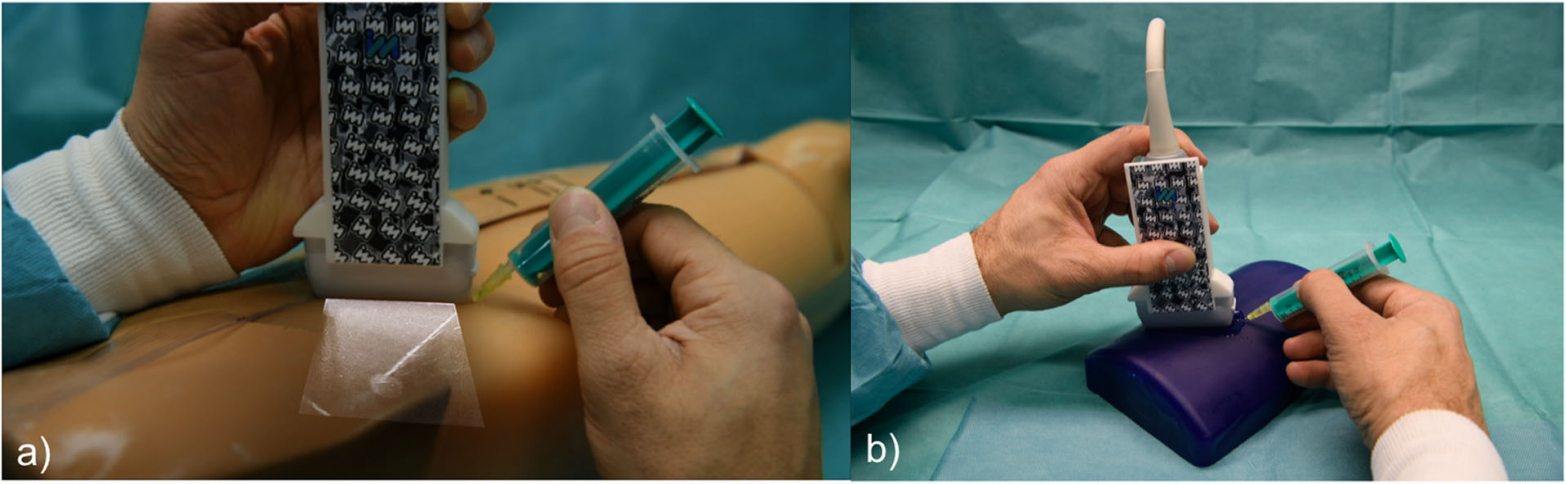
Demonstration of the study setup, using augmented reality in situ ultrasound to puncture the soft tissue lesions in the leg phantom with operator view (**a**) or the vessel in the blue phantom without operator view (**b**)

The punctures were performed using a 20-gauge yellow needle (7-cm long) in a leg phantom (leg model with soft tissue biopsy insert, blue phantom, CAE Healthcare, Synmedic AG, Zurich, Switzerland) including 20 soft tissue lesions of varying lesions ranging from 4 to 11 mm in diameter. The leg phantom size was 81 × 20 × 20 cm; the weight 11.5 kg. Ten of those lesions were marked and numbered, ensuring that each operator punctured the same lesions in the same order (Figs. 2 and 3a). Further, each operator had to puncture the same vessel (6 mm in size) in a vessel phantom (Blue phantom, CAE Healthcare, Synmedic AG, Zurich, Switzerland) ten times (Fig. 3b). These punctures were initially performed using AR *in situ* US, and > 4 weeks later, each operator repeated the punctures using the conventional US, in order to avoid a possible training effect after the first puncture session. The location of the needle tip was documented after each puncture by an experienced radiologist in US in the leg phantom and verified by fluid aspiration in the vessel phantom. Time to puncture and number of needle passes as well as location (correct or incorrect) of needle tips were documented for each puncture using the Redcap software (Vanderbilt University Medical Center, Version 8.11.5, Nashville, USA) [18].

### Data presentation

Descriptive statistic was performed using the software PRISM (Version 8, Graphpad software, La Jolla (CA), USA). Medians and ranges were used to report the non-parametric data. By purpose, being this a proof-of mechanism phantom study for which sample size was not preliminarily estimated, and based on consultation with the statistician, we did not perform statistical testing for significance.

## Results

### Conventional US *versus* AR *in situ* US

**Fig. 4:**
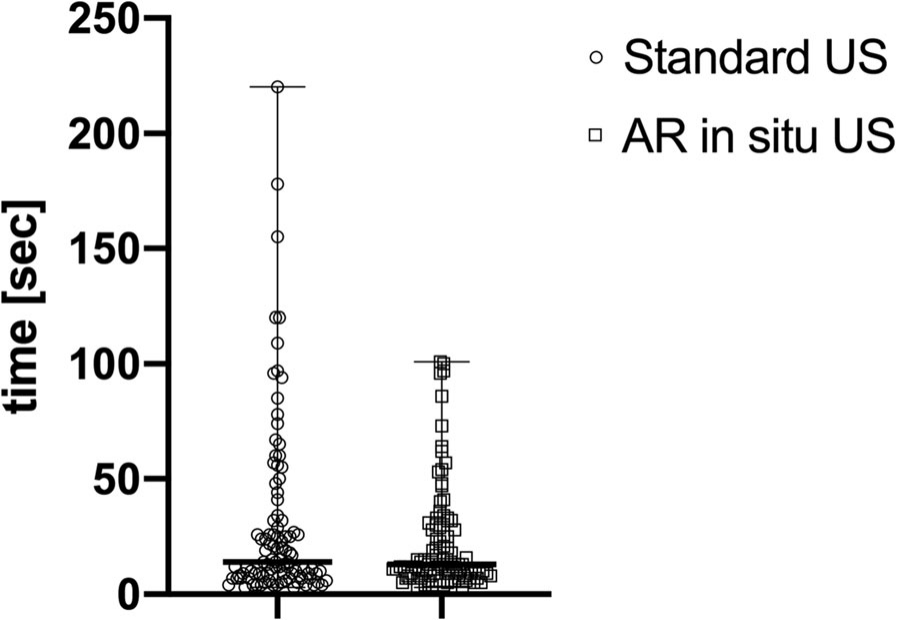
Differences of time to target puncture of all operators using the standard ultrasound *versus* augmented reality *in situ* ultrasound technique

**Fig. 5:**
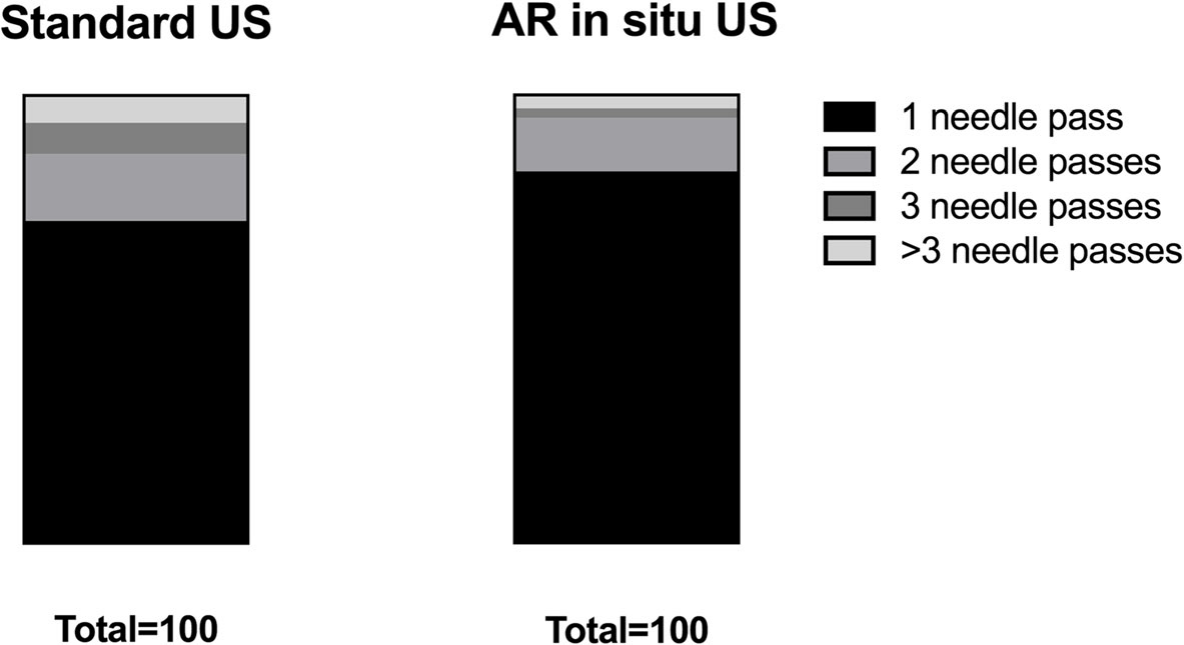
Differences of number of needle passes to target puncture of all operators using the standard ultrasound *versus* augmented reality *in situ* ultrasound technique

The time to puncture was overall reduced using AR *in situ* US (13 s [3–101], median [range]) compared to the conventional US technique (14 s [3–220] (Fig. 4). The number of needle passes were also reduced using AR *in situ* US (1 [1–4], median [range]) compared to conventional US (1 [1–8], Fig. 5). Achieving correct location eventually was similar (0.6 [0.2–1.5] odds ratio [95% confidence interval]) using either technique (AR *in situ* US, 90% (90/10); conventional US, 94% (94/6)).

### Inter-operator performances

**Fig. 6:**
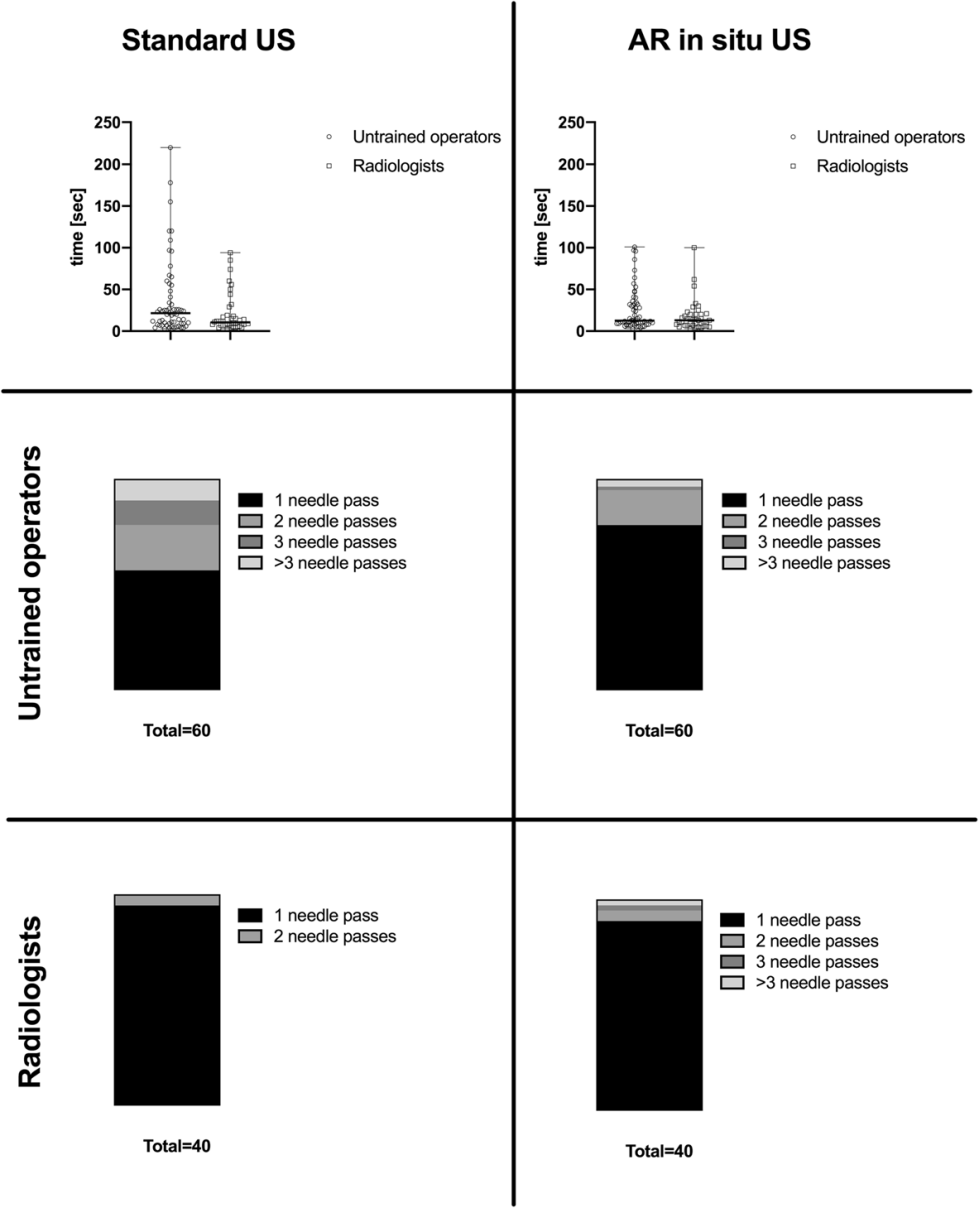
Experience-based differences using the two different modalities (standard ultrasound *versus* augmented reality *in situ* ultrasound technique). Differences in time and number of needle passes to target puncture in untrained operators *versus* radiologists using standard ultrasound *versus* augmented reality *in situ* ultrasound technique

Experienced radiologists were faster and needed less needle passes when performing the punctures with the conventional US (time 10.5 s [3–94]; needle passes, 1 [1, 2]) compared to the untrained operators (time 21.5 s [3–220]; needle passes, 1 [1–8]) (Fig. 6). When using AR *in situ* US, the difference between experienced and untrained operators was smaller (time 13 s [3–100] *versus* 12.5 s [3–101]; needle passes, 1 [1–4] *versus* 1 [1–4]; Fig. 6). The differences between untrained operators *versus* radiologists were more pronounced in the leg phantom. The differences of time and counted needle passes to puncture the targets within the different phantom types are summarised in Table 1.

**Table 1:** Time and counted needle passes using standard ultrasound *versus* augmented reality *in situ* ultrasound in the leg phantom and vessel phantom in untrained and trained operators

	Standard US leg phantom		Standard US vessel phantom		AR *in situ* US leg phantom		AR *in situ* US vessel phantom	
	Untrained	Trained	Untrained	Trained	Untrained	Trained	Untrained	Trained
Time (s), median [range]	33 [7–220]	18.5 [8–94]	10.5 [3–60]	7 [3–15]	32 [7–101]	16.5 [5–100]	9 [3–34]	9 [3–25]
Counted needle passes, median [range]	2 [1–8]	1 [1–2]	1 [1–6]	1 [1–1]	1 [1–4]	1 [1–4]	1 [1–2]	1 [1–1]

## Discussion

Ultrasound-guided punctures might be challenging and require training and expertise to be reliable and reproducible. We investigated if the combination of US with AR technology achieving an AR *in situ* US view with the US image as hologram, displayed at the exact anatomic location in reality, would reduce inter-operator variability in performance, by simplifying the challenge of spatial orientation. As expected, we observed a relevant difference in performance of untrained *versus* experienced operators using conventional US-guided punctures. This difference decreased when enforcing untrained operators with AR *in situ* US technology. The operator dependency is taught to be caused by differences in ability for spatial orientation. We assume that AR *in situ* US simplifies spatial orientation for US-guided interventions, since the US image is directly superposed on the anatomy in a 1:1 ratio and therefore the operator can directly aim the tip of the needle into the US image.

Not only the operator dependent differences were diminished, but also intraindividual differences, such as AR *in situ* US saved time and needle passes thorough all operators. We think this can simply be explained as the time turning the head to the monitor’s image can be spared. Therefore, it is plausible that untrained operators needing more corrections also needed to turn their head more often to the monitor and thus needing more time in total compared to the radiologists. The differences between untrained operators *versus* radiologists were more pronounced in the leg phantom, as the punctured targets were more difficult to puncture—the targets to puncture were in different depth within the soft tissue and deeper below the surface compared to the vessel phantom, where the blood vessel was a few centimetres below the surface and was for all punctures at the same height.

There are only few comparable US studies published using a similar technique with tracking the US probe and overlying the US image to its anatomical position [19, 20]. The study of Rosenthal et al. [19] found that operators performed better using the AR *in situ* technology; however, only needle placement accuracy were compared between the two methods (AR US *versus* standard US). Maas et al. [20] used the same technique to visualise the foetus on a portable device for the parents. Other studies using AR image overlay techniques for punctures needed manual alignment of the hologram, which was the main disadvantage [10]. The manual alignment is no longer needed with the here introduced method, as the image position is directly displayed at the anatomical position, that further increases precision of punctures, as malalignment of holograms or malalignments because of patient motions between image acquisition and puncture is negligible, in contrast to other studies [10, 14, 21, 22].

Other advantages here not investigated could be the direct view to the patient at all times with AR *in situ* US, which could be important in case of patient motion, particularly considering other potential application such as breast mass biopsies, liver biopsies, and also injections for magnetic resonance arthrography or therapeutic joint injections. One other advantage of the use of AR *in situ* US using a head-mounted device is the better mobility and easier handling of the entire system. Further, even if we investigated feasibility with a linear probe, other probes (independent of the manufacturer) could be easily adaptable to the here presented technology. Also, the cost of head-mounted devices has decreased over the last decades rapidly, making a potential cost-effectiveness of such system realistic in future.

Since this is the first study describing this potential breakthrough in US imaging, comparable study methodologies were difficult to identify. Two different phantoms, namely a leg phantom with soft tissue lesions and a phantom with vessels, were used to simulate two common clinical applications. Certainly, this is one of the main limitations of the study. External validity for clinical situations is not claimed and is subject of current investigations at our institution. We identified a further limitation that was difficult to address in designing this phantom study: repetitive interventions allow a learning curve and could have biased the measured outcomes. Therefore, first, the initial punctures were all performed using AR *in situ* US; on a separate day (> 4 weeks later), the same punctures were repeated using the conventional US. With this, we tried to reduce the difference of operator-dependent performance as a result of a potential learning curve.

In conclusion, this proof-of mechanism phantom study showed that AR *in situ* US technology could be a potential breakthrough for US-guided interventions by simplifying the operator’s spatial orientation, with a potential for reducing the inter- and intra-operator variability.

## Abbreviations

AR: Augmented reality
US: Ultrasound
